# Accounting for single center effects in systematic reviews cannot be overlooked

**DOI:** 10.1186/s13054-017-1804-0

**Published:** 2017-09-15

**Authors:** Bruno Adler Maccagnan Pinheiro Besen, Marcelo Park, Antonio Paulo Nassar

**Affiliations:** 1 Medical Intensive Care Unit, Medical Emergencies Discipline, Hospital das Clínicas, University of São Paulo Medical School, São Paulo, Brazil; 2 Intensive Care Unit, Hospital da Luz, São Paulo, Brazil; 3 Intensive Care Unit, A.C. Camargo Cancer Center, São Paulo, Brazil

We read with interest the recently published systematic review and meta-analysis on timing of renal replacement therapy (RRT) in cardiac surgery patients [1]. However, we are worried with the presented results and the possible impact on clinical practice of this study.

The author’s have included two studies that were not related to the inclusion criteria. Lange et al.’s study [2] was merely descriptive and assessed risk factors for worse outcomes, with no mention of early or late initiation of RRT. Li et al.’s study [3] compared different doses of RRT and reported no differences in mean time (h) to initiation of RRT. Furthermore, the authors included a study in the analysis that was not related to cardiac surgery [4].

In our opinion, these downsides are a threat to the internal validity of the systematic review and preclude any meaningful interpretation of results. Even more so, the author’s have not reported a subgroup analysis to investigate single-center effects, which are known to bias results from meta-analysis [5]. To address this, we have performed a random-effects meta-analysis excluding studies that were not related to the systematic review’s original inclusion criteria and with a subgroup analysis to investigate single center effects (Fig. 1). When considering only multicenter trials, the point estimate is pulled to the null and the results are neutral. This is unsurprising since recent systematic reviews comprising mixed populations of critically ill patients have not shown any beneficial effect when pooling results across randomized clinical trials at low risk of bias.

**Fig. 1:**
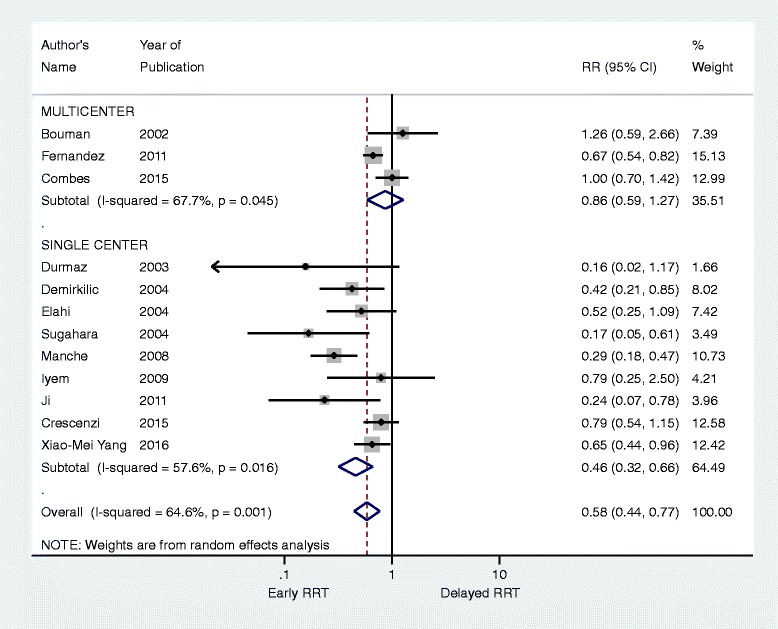
Forest plot, stratified according to the number of centers of the original studies. Studies not fulfilling inclusion criteria of the systematic review’s study question [1] were not included in this analysis. *RRT* renal replacement therapy, *RR* risk ratio, *CI* confidence interval

Therefore, we believe the results of this meta-analysis should be cautiously interpreted before widespread adoption of early RRT as a standard practice, pending the results of adequately designed higher-quality randomized clinical trials.

## Additional files

Additional file 1: Forest plots showed early RRT initiation group decreased 28-day mortality in patients with AKI after cardiac surgery. (PDF 37 kb)

Additional file 2: The subgroup analysis showed early RRT initiation within 24 hours was associated with low mortality in patients with AKI after cardiac surgery. (PDF 45 kb)

## Abbreviations

RRT: Renal replacement therapy
