# Supplementary material for: Accounting for single center effects in systematic reviews cannot be overlooked
Source: Crit Care. 2017 Sep 15;21:241. doi: 10.1186/s13054-017-1804-0 (PMC5602911; doi:10.1186/s13054-017-1804-0)
Supplement: Supplementary file 1 — Forest plots showed early RRT initiation group decreased 28-day mortality in patients with AKI after cardiac surgery. (PDF 37 kb) [file 13054_2017_1804_MOESM1_ESM.pdf]

Study or Subgroup	Early Events	Early Total	Late Events	Late Total	Weight	Odds Ratio M-H, Random, 95% CI
Bouman 2002	11	35	9	36	9.2%	1.38 [0.49, 3.88]
Combes 2015	40	112	40	112	12.3%	1.00 [0.58, 1.73]
Crescenzi 2015	28	46	10	13	7.1%	0.47 [0.11, 1.93]
Demirkilic 2004	8	34	15	27	8.9%	0.25 [0.08, 0.74]
Durmaz 2003	1	21	7	23	4.2%	0.11 [0.01, 1.03]
Elahi 2004	8	36	12	28	8.9%	0.38 [0.13, 1.13]
Fernandez 2011	54	101	82	102	11.9%	0.28 [0.15, 0.52]
Iyem 2009	5	95	6	90	8.1%	0.78 [0.23, 2.64]
Ji 2011	3	34	9	24	7.0%	0.16 [0.04, 0.68]
Manche 2008	14	56	13	15	6.2%	0.05 [0.01, 0.26]
Sugahara 2004	2	14	12	14	4.4%	0.03 [0.00, 0.23]
Xiao-Mei Yang 2016	20	59	80	154	11.9%	0.47 [0.25, 0.89]
**Total (95% CI)**		**643**		**638**	**100.0%**	**0.35 [0.20, 0.59]**
Total events	194		295			

Heterogeneity: Tau² = 0.52; Chi² = 34.09, df = 11 (P = 0.0003); I² = 68%
Test for overall effect: Z = 3.87 (P = 0.0001)

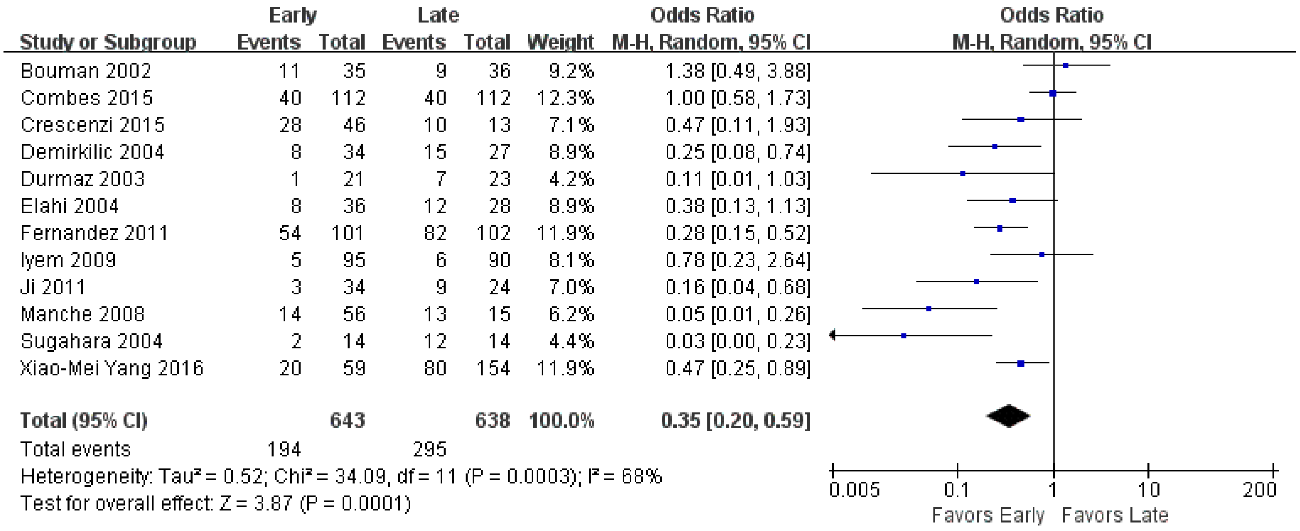

Additional file 1: Forest plots showed early RRT initiation group decreased 28-day mortality in patients with AKI after cardiac surgery.
