# Supplementary material for: Accounting for single center effects in systematic reviews cannot be overlooked
Source: Crit Care. 2017 Sep 15;21:241. doi: 10.1186/s13054-017-1804-0 (PMC5602911; doi:10.1186/s13054-017-1804-0)
Supplement: Supplementary file 2 — The subgroup analysis showed early RRT initiation within 24 hours was associated with low mortality in patients with AKI after cardiac surgery. (PDF 45 kb) [file 13054_2017_1804_MOESM2_ESM.pdf]

Study or Subgroup	Early Events	Early Total	Late Events	Late Total	Weight	Odds Ratio M-H, Random, 95% CI
**2.1.1 12h**						
Bouman 2002	11	35	9	36	10.4%	1.38 [0.49, 3.88]
Crescenzi 2015	28	46	10	13	8.2%	0.47 [0.11, 1.93]
Ji 2001	3	34	9	24	8.0%	0.16 [0.04, 0.68]
Manche 2008	14	56	13	15	7.2%	0.05 [0.01, 0.26]
Sugahara 2004	2	14	12	14	5.2%	0.03 [0.00, 0.23]
**Subtotal (95% CI)**		**185**		**102**	**39.1%**	**0.20 [0.05, 0.81]**
Total events	58		53			
Heterogeneity: Tau² = 2.03; Chi² = 18.87, df = 4 (P = 0.0008); I² = 79%						
Test for overall effect: Z = 2.24 (P = 0.02)						
**2.1.2 24h**						
Combes 2015	40	112	40	112	13.5%	1.00 [0.58, 1.73]
Demirkilic 2004	8	34	15	27	10.0%	0.25 [0.08, 0.74]
Elahi 2004	8	36	12	28	10.1%	0.38 [0.13, 1.13]
Xiao-Mei Yang 2016	20	59	80	154	13.0%	0.47 [0.25, 0.89]
**Subtotal (95% CI)**		**241**		**321**	**46.6%**	**0.52 [0.28, 0.95]**
Total events	76		147			
Heterogeneity: Tau² = 0.21; Chi² = 7.10, df = 3 (P = 0.07); I² = 58%						
Test for overall effect: Z = 2.14 (P = 0.03)						
**2.1.3 48h**						
Durmaz 2003	1	21	7	23	5.0%	0.11 [0.01, 1.03]
Iyem 2009	5	95	6	90	9.3%	0.78 [0.23, 2.64]
**Subtotal (95% CI)**		**116**		**113**	**14.3%**	**0.37 [0.06, 2.37]**
Total events	6		13			
Heterogeneity: Tau² = 1.07; Chi² = 2.30, df = 1 (P = 0.13); I² = 56%						
Test for overall effect: Z = 1.05 (P = 0.30)						
**Total (95% CI)**		**542**		**536**	**100.0%**	**0.35 [0.19, 0.64]**
Total events	140		213			
Heterogeneity: Tau² = 0.61; Chi² = 31.33, df = 10 (P = 0.0005); I² = 68%						
Test for overall effect: Z = 3.43 (P = 0.0006)						
Test for subgroup differences: Chi² = 1.55, df = 2 (P = 0.46), I² = 0%						

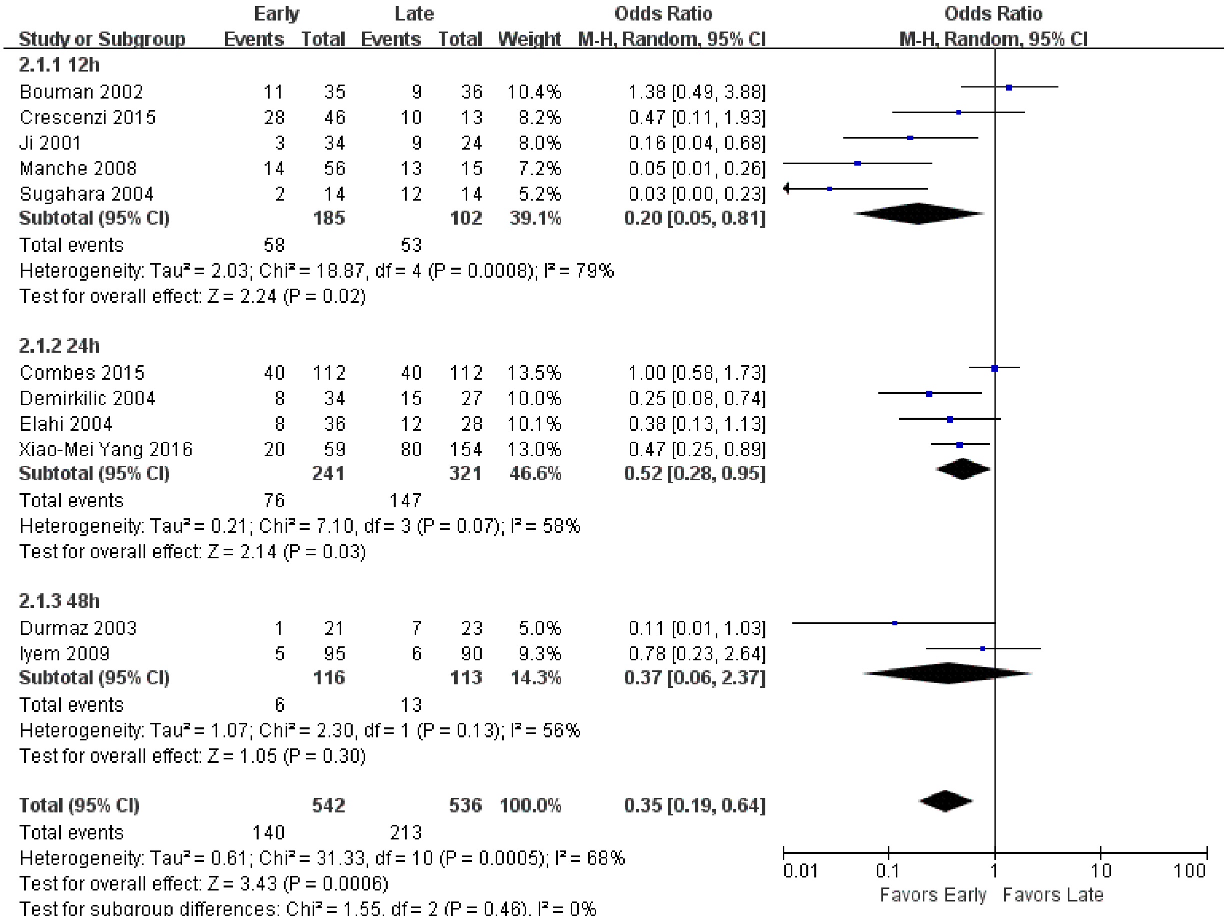

Additional file 2: The subgroup analysis showed early RRT initiation within 24 hours was associated with low mortality in patients with AKI after cardiac surgery.
