# Supplementary material for: Accounting for single center effects in systematic reviews cannot be overlooked
Source: Crit Care. 2017 Sep 15;21:241. doi: 10.1186/s13054-017-1804-0 (PMC5602911; doi:10.1186/s13054-017-1804-0)
Supplement: Related texts

# Reply: Authors’ response

**Authors:** Gaosi Xu

We are grateful to Professor Besen for his comments. First of all, we agree it is preferable for a meta-analysis to include original multi-centered randomized controlled trials; however, well-designed studies such as these are unfortunately lacking in cardiac surgery. Second, it can be seen in Fig. 1 that the heterogeneity of the multi-centered trials included is moderate (67.7%), and that only three trials are studied. Third, in Li et al.’s study [3], one of the purposes of the research was to investigate timing to RRT initiation, and the indication for RRT was urine output < 240 mL/12 h regardless of other symptoms. Li et al.’s study compared different doses of RRT as well as timing to RRT initiation, and the high-dose group received early RRT [3]. In other words, the study indicated that an early higher continuous veno-venous hemofiltration dose was associated with better in-hospital and long-term survival [3]. As for the study by Kleinknecht et al. [4], we think that this can be included in our meta-analysis as patients who suffered from acute kidney injury in post-cardiac surgery are included. Fourth, we performed a meta-regression in our meta-analysis [1] but did not find the sources of heterogeneity, so no other subgroup analysis was done. Last but not least, the results of the analysis of early versus late RRT initiation (Additional file 1) and subgroup analysis of the timing to early RRT initiation (Additional file 2) are consistent with our original results even after exclusion of these three studies [2–4]. The results are therefore that early RRT initiation decreases 28-day mortality, especially when started within 24 h, in patients with severe acute kidney injury after cardiac surgery.
